# Web Real-Time Communications-Based Unmanned-Aerial-Vehicle-Borne Internet of Things and Stringent Time Sensitivity: A Case Study

**DOI:** 10.3390/s25020524

**Published:** 2025-01-17

**Authors:** Agnieszka Chodorek, Robert Ryszard Chodorek

**Affiliations:** 1 Department of Applied Computer Science, Faculty of Electrical Engineering, Automatic Control and Computer Science, Kielce University of Technology, Al. 1000-lecia P.P. 7, 25-314 Kielce, Poland; 2 Institute of Telecommunications, Faculty of Computer Science, Electronics and Telecommunications, AGH University of Krakow, Al. Mickiewicza 30, 30-059 Krakow, Poland

**Keywords:** IEEE 802.11ac, internet of things, low latency, real-time transmissions, unmanned aerial vehicle, WebRTC, WebSocket

## Abstract

The currently observed development of time-sensitive applications also affects wireless communication with the IoT carried by UAVs. Although research on wireless low-latency networks has matured, there are still issues to solve at the transport layer. Since there is a general agreement that classical transport solutions are not able to achieve end-to-end delays in the single-digit millisecond range, in this paper, the use of WebRTC is proposed as a potential solution to this problem. This article examines UAV-borne WebRTC-based IoT in an outdoor environment. The results of field experiments conducted under various network conditions show that, in highly reliable networks, UAV and WebRTC-based IoT achieved stable end-to-end delays well below 10 ms during error-free air-to-ground transmissions, and below 10 ms in the immediate vicinity of the retransmitted packet. The significant advantage of the WebRTC data channel over the classic WebSocket is also demonstrated.

## 1. Introduction

Unmanned aerial vehicles (UAVs) are currently one of the fastest developing multi-role carrier technologies. These ubiquitous devices now have a multitude of economic, commercial, leisure, military, and academic uses [1], and their uses range from individuals flying them for recreation to large commercial package and medical supply companies [2]. They can be used to transport parcels and people between locations [3,4,5]. Equipped with on-board cameras and Internet of Things (IoT) systems, UAVs are used to monitor pollution [6,7], weather [8,9], road traffic [10], and crop production [11,12]. An important part of these UAV applications is the communication and computing support, including a flying range extender [13], a flying router [13,14], and a flying computer for aerial mobile edge computing (AMEC) purposes [15].

These and other UAV applications can be time-sensitive in a broad sense, i.e., they may have arbitrary time constraints imposed. These can be relatively large if they are related to the delivery of parcels or people. Such deliveries may have to be completed within a specific time window [3,4] or as soon as possible. It is estimated that, in a large city, the time needed for transporting parcels or people by air may be a few dozen percent shorter than the time for land transport [5]. Time constraints may also be relatively small when they concern the provision of real-time or near-real-time information: either to detect and locate the source of pollution [6,7], or for disaster response purposes [13,14]. The need for real-time information may also arise in the case of observations of weather [8,9], crop production [11,12], and road traffic [10], etc., if data are sent from the UAV to a ground station, where they are used for analysis, real-time visualization, and decision-making.

Time-sensitive UAV-IoT applications may also involve collecting and processing data from sensors located in a given area. For example, the integration of UAVs with the internet of medical things (IoMT) was reported in [15]. This UAV-enabled system implements AMEC functionality. In the proposed solution, communication delays were reduced from a range of 17 ms to 30 ms to a range of 12.5 ms to 24 ms. The freshness of data is expressed in the so-called age of information (AoI), i.e., the time that has passed since the generation of the most recently received data [16]. AoIs known from the literature include UAV flight time, hover time, and maintenance time, and range from less than 600 s to less than 1800 s [16] or from almost 1400 s to less than 2200 s [17], with low-AoI systems starting with an AoI of 70–80 s [18,19]. AoI improvement methods are based on optimizing the UAV trajectory [16,17,18,19], and the transmission delay for such large times is negligible.

Currently, the main challenge in the field of wireless communication with UAVs is time-sensitive applications that require low latency, defined as end-to-end delays measured in single-digit milliseconds at the application level. Examples of such applications are presented in Table 1. The traffic generated by these applications is deterministic, meaning hard real-time with no jitter, or non-deterministic, where low jitter can be observed. A high reliability of transmission is required, and in the case of deterministic traffic, ultra-high reliability is needed. This approach breaks with the classic division of telecommunications traffic into elastic and inelastic, where only inelastic traffic had to meet stringent time requirements and only elastic traffic had to be characterized by a high transmission reliability [20].

It is important to note here that using a low-latency network does not guarantee low end-to-end delays at the application level. This state of affairs is blamed on the mechanisms of classic transport protocols, which are unable to effectively meet the requirements of low delays [23]. Another problem is the socket application programming interface (API) for these protocols, which is too low-level, simple, and inflexible [23]. The authors believe that a solution to the above problems, at least in the case of time-sensitive UAV-borne IoT, could be the use of web real-time communications (WebRTC), which, as the name suggests, provides native real-time communication on the Web. The World Wide Web Consortium (W3C) in the document in [26] announced the general need for building a WebRTC-based IoT. Requirement N15 included in [26] states that a WebRTC-based IoT should be able to provide low and consistent latency under varying network conditions.

### Main Contributions and Organization of This Paper

In our previous paper, we proposed a WebRTC-based application capable of operating like the classic IoT [27], intended for use in UAV-borne monitoring systems. This application was a part of our UAV- and WebRTC-based open universal framework [28]. In this paper, we present the results of field experiments aimed at verifying whether, and to what extent, a UAV-borne IoT based on the current WebRTC standard is able to provide low and consistent latency under varying network conditions. The main contributions of this paper are as follows:

- Supplementing the application in [27], working as an element of the framework [28], with high-resolution time measurement and timer synchronization procedures.
- Carrying out delay measurements at the level of the transport protocol and at the level of the web logical channel during air-to-ground IoT transmissions under varying network conditions, and then performing a statistical analysis of these delays.
- For the completeness of the results, a comparison of the obtained results with the results obtained for IoT transmission via a classic web logical channel, i.e., WebSocket, in the same circumstances.

The rest of this paper is organized as follows: Section 2 analyzes related work. Section 3 discusses the materials and methods used during the experiments. Section 4 describes the field experiments, including post-selection of the measurement series for further analysis. Section 5 presents and discusses the measures of the location of the selected series of end-to-end delays, while Section 6 compares the transmissions carried out with the use of WebRTC and WebSocket in terms of the measures of location, as well as the measures of variation derived from these measures of location. Section 7 summarizes our experiences.

## 2. Related Work

While Section 1 provides a broad background, Section 2 discusses both real-time alternatives and the authors’ prior application solutions that formed the basis of this paper. The review of existing solutions covers time-sensitive applications that generate non-deterministic traffic. Although real-time transmission is usually associated with multimedia streaming (as are IoT real-time transmissions [29]), the paper only discusses the transmission of non-media data, usually data coming from sensors. The discussion focuses on aspects of the transport layer, i.e., transport protocols and interfaces. The criterion for selecting literature was the various non-media real-time transmission techniques found in the literature, preferring papers that explicitly provided transmission times in a local area network. Most references concern IoT communication between UAVs and ground stations.

Non-deterministic traffic generated by time-sensitive applications is most often transmitted air-to-ground using wireless local networks (WLANs), usually built using the Institute of Electrical and Electronics Engineers (IEEE) 802.11 standard [27,28,30,31,32,33,34,35,36,37,38,39,40], but also using standards for broadband cellular networks: the long-term evolution (LTE) standard [40,41], also known as the fourth-generation (4G) technology standard, and the fifth-generation (5G) technology standard [15,30,40]. Among IEEE 802.11 [42] networks, popular versions of the physical layer are used, such as 802.11g [31,32] and 802.11n [33,34,35,40], as well as the 802.11p version intended for the vehicular environment [30]. The works [27,28] employed the 802.11ac version, which is able to provide latencies below 10 ms, including providing handover latencies below 10 ms thanks to the fast roaming service [43].

The 802.11 standard was also used in [44], where a time-sensitive application, intended to work on board a UAV, was tested using a laptop and an unmanned ground vehicle (UGV). Another time-sensitive application in which the sensor system was carried on board a UGV was presented in [45]. The network used in [45] was based on software-defined radio (SDR) working with software-defined networking (SDN). In [46], a stationary robot communicated via both 802.11 WLAN and evolved high-speed packet access (HSPA+), also known as the 3.75G technology standard. As a side note, refs. [44,46] also tested transmissions between stationary end systems over longer distances using the public infrastructure of an Internet service provider (ISP). These are not the subject of this article, because current networks, including those built using 5G technology [47], are only able to provide low-latency services locally, using dedicated low-latency solutions with limited range.

Time-sensitive applications typically send sensor data using the classic transmission control protocol (TCP), which provides reliable congestion- and flow-controlled transmission. Applications use the TCP transport protocol directly [41] or via the WebSocket web logical channel [30,34,35,36,38,39,44,45,46]. The works in [31,32,33] used a multipath version of the TCP protocol, i.e., the multipath transmission control protocol (multipath TCP or MPTCP), which allows multi-homed senders to increase transmission efficiency. The use of MPTCP has been shown to reduce latency under certain conditions [48], although the protocol is sensitive to path asymmetry, especially if the paths are built with different technologies (e.g., 4G and 802.11) [49]. In the works in [31,32], MPTCP was modified to meet the requirements of time-sensitive networking (TNS). In [33], to deal with network stability in the face of high UAV mobility, the MPTCP scheduling algorithm was modified.

While the TCP protocol has been typically used for transmission of non-media data, another classic transport protocol, i.e., the user datagram protocol (UDP), is used for audio/video transmission, due to its simple structure and mechanisms reduced to an absolute minimum, including the lack of window-based control. Currently, the UDP is most often used as an underlay protocol for other transport protocols, where it occupies a lower sublayer of the transport layer. The most popular solution is to use the real-time transport protocol (RTP) in the upper sublayer. A RTP/UDP protocol stack is typically used for multimedia communications. This solution is also used in the WebRTC video channel. In [27,28,34,35,46], a WebRTC video channel was used to transmit video from a camera. In [44], it was used to transmit data from lidar. WebRTC uses the RTP protocol implemented in a WebRTC-capable browser and the UDP protocol implemented in an operating system.

UDP can also be used as a transport protocol for transmitting non-media data. In [41], a low-latency reliable transmission (LRT) application layer protocol operating directly over UDP was proposed to reduce the delay during ground-to-UAV transmission through a cellular network. In the abovementioned work [31], control data were sent via the UDP protocol, while the remaining data were sent via the MPTCP protocol. The WebRTC data channel used the stream control transmission protocol (SCTP) over UDP. To ensure reliable transmission, the SCTP uses error control and congestion control mechanisms similar to those of TCP. In the works in [27,28,46], the SCTP protocol was used to transmit data from sensors. Similarly to the RTP, the SCTP is always implemented in a WebRTC-capable browser, regardless of the operating system’s implementation of the SCTP. This is due to the need to use the new version of the SCTP standard intended for WebRTC [50]. However, the implementation of the SCTP in the operating system can also be used for IoT data transmission [37]. The UDP transport protocol implemented in the operating system is also the basis for the quick UDP internet connections (QUIC) protocol [51], initially intended for web applications and now proposed for low-latency communication in the next-generation IoT [52]. The papers in [38,39] presented the results of evaluations of a real IoT transmitted using the QUIC in an emulated [38] or simulated [39] wireless environment.

Applications, including web browsers, use the TCP and UDP transport protocols implemented in the operating system, communicating with them via the socket interface. In [31,32,33], applications communicated with the MPTCP protocol in the operating system via a classic stream socket. The WebSocket web logical channel uses the TCP protocol in the operating system, also communicating with it via a stream socket. Applications that send data via WebSocket, such as [30,34,44,45], use the high-level WebSocket API. WebRTC offers separate web logical channels for media and non-media transmission, each of which is associated with a separate high-level API used by WebRTC applications such as [27,28,34,35,44,46]. The RTP and SCTP protocols are implemented in WebRTC-capable browsers and communicate with the UDP protocol in the operating system via classic datagram sockets. WebRTC does not use the SCTP in the operating system and therefore does not use an SCTP socket.

A comparison of related work is presented in Table 2. Our WebRTC-based UAV-borne IoT application is presented in [27,28]. In this work, data were not transmitted in the web of things (WoT) architecture [53], using an intermediate server, but in the classic IoT manner, using a peer-to-peer WebRTC architecture. In [44], transmissions of lidar data via the WebRTC video channel and via Websocket were compared. In [34,35], IoT data were transmitted via Websocket, and only video was transmitted via WebRTC. In [46], WoT data were transferred from a robot to a WoT server over WebSockets, and then from the server to the recipient over WebRTC. In [40], WebRTC data channel was used to control a UAV and transmit telemetry. The remaining papers did not use WebRTC. In [30,36,45], only a WebSocket logical channel was used, while, in [15,31,32,33,37,38,39,41], a web logical channel was not used at all.

WebRTC applications are web-based equivalents of classic, standalone multimedia applications based on the session initiation protocol (SIP). The management plane protocol stack [54] and the production plane protocol stack for media streaming [55] are similar to their legacy SIP architecture counterparts. WebRTC applications are loaded from web servers as part of web pages and use web browsers as run-time environments. This makes them highly portable and secure, as detected browser vulnerabilities are eliminated on an ongoing basis. What distinguishes WebRTC from other web techniques, such as WebSocket, is its dual protocol stack, the idea of which was taken from the SIP architecture. As an effect, WebRTC can be used to transmit both media streams and non-media flows. Streams and flows are cryptographically protected and congestion-controlled. Since both the media stream and non-media flow use TCP-friendly congestion control, in the event of poor network conditions, data are protected at the expense of video [56]. If necessary, WebRTC applications can use more sophisticated streaming media congestion control methods, such as RTP translators or simulcast, and both RTP streams and SCTP flows can use differentiated services (DiffServ) to ensure quality of service (QoS).

## 3. Materials and Methods

This section introduces the flying monitoring system used in the field experiments, highlighting the time-sensitive aspects; defines the end-to-end delays measured at both the logical channel level and transport protocol level; shows the method for creating of a series of end-to-end delays; and finally describes the extreme values and measures of location calculated from these series.

### 3.1. System

In all experiments, a flying monitoring system built on the basis of the framework in [28] was used. Structurally, the system consists of an air station and a ground station, and functionally of an IoT system and an IoT carrier. The air station was an unmanned quadcopter, operating as the IoT carrier, with an IoT system on board, i.e., environmental sensors connected to a single-board computer (SBC) Raspberry Pi 4 Model B running the authors monitoring application. The environmental sensors included four weather sensors previously used to build a mobile weather station [9] and a gas sensor used in a pollution monitoring system [7], which allowed for the reuse of existing sensor-dependent code. The monitoring application was written in the JavaScript language as part of a web page, and its runtime environment was the Chromium browser in headless mode and run on the Raspberry Pi OS operating system. The authors’ analysis of the Chromium browser implementation showed that the browser has a limited send buffer, which allows transmissions to reduce buffering times, and the stream socket was set to disable Nagle’s algorithm, so there was no need to additionally set these parameters.

The monitoring application included the WebRTC video service, positioning service, and sensor service. The WebRTC video service was built as a classic WebRTC video application. The positioning service and the sensor service were built in a browser-driven manner [27], typical for IoT systems. In the experiments presented in this paper, the video service was turned off, the positioning service sent its data to the sensor service, and only the sensor service sent its data to the ground. Data from sensor service were transmitted in message queuing telemetry transport (MQ telemetry transport, or MQTT) messages bearing the MQTT topic, which identified each datum. Example topics used in the experiments and the method of creating them were described in the authors previous paper [7]. MQTT messages were transmitted over a web logical channel, using both the WebRTC data channel and the WebSocket. In the latter case, to improve the time properties of the TCP, the PUSH option was set in each TCP packet carrying the MQTT message, which means immediate pushing of the received data to the application. Because the correct operation of the monitoring application required that the central processing unit (CPU) always had a sufficient reserve of resources, this had to be monitored during the performance of all tests.

**Figure 1:**
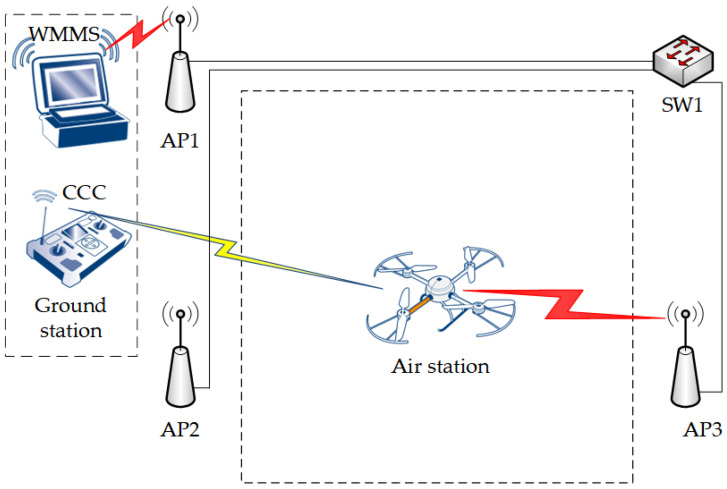
The testbed.

Unlike the air station, which was one device, the ground station was divided into two separate devices (Figure 1): the command and control console (CCC), and the WebRTC multimedia and monitoring station (WMMS). The CCC was used to pilot the IoT carrier. It was connected to the UAV via the control network (yellow lightning in Figure 1). The WMMS is designed for IoT purposes. It was connected to the monitoring software via the IEEE 802.11ac production network (red lightning in Figure 1), which was built as a heterogeneous extended service set (ESS). As an effect, the transmission between the air station and the WMMS was carried out both in a wireless environment and in a mixed wired/wireless one, in which the access points were connected to each other via a gigabit Ethernet (IEEE 802.3ab) network. The intermediate devices used in the ESS were NETGEAR Nighthawk X4 R7500 AC2350 access points (AP1, AP2, and AP3) and an HP 3500-24G-PoE+ yl Switch (SW1). The AP1 and the SW1 were placed close to the corners of a rectangular 70 m × 70 m parking lot, which was the test area. The AP2 and the AP3 were located at 50 m from the AP1 and the SW1, respectively.

### 3.2. Series of End-to-End Delays

During the flights, time parameters were collected, both at the air station and at the ground station. These parameters were used to determine a pair of delays: one at the transport level, the other at the level of the web logical channel.

**Definition** **1.** *The end-to-end delay $d_{i}^{t}$ of the i-th IoT datum transmitted between the air station and the ground station, measured at the transport level, is defined as*

(1) $$d_{i}^{t}=t_{i}^{t}-t_{i}^{ilc}$$

*where $t_{i}^{t}$ is the reception time from the transport protocol and $t_{i}^{ilc}$ is the entry time into the logical channel.*

**Definition** **2.** *The end-to-end delay $d_{i}^{t}$ of the i-th IoT datum transmitted between the air station and the ground station, measured at the logical channel level, is defined as*

(2) $$d_{i}^{lc}=t_{i}^{olc}-t_{i}^{ilc}$$

*where $t_{i}^{olc}$ is the reception time from the logical channel.*

In the case of transmissions carried out over the WebRTC Data Channel, the times $t_{i}^{ilc}$, $t_{i}^{t}$ and $t_{i}^{olc}$ were measured for each transmitted IoT datum, where *i* was the sequence number of this datum. From these times, the delays $d_{i}^{t}$ and $d_{i}^{lc}$ were then calculated according to Formulas (1) and (2), respectively. The difference between corresponding delays resulted from the processing of the payload of the SCTP packet (MQTT message) placed in the receive buffer of the logical channel. In the case of transmissions conducted over the WebSocket logical channel, performed for comparison purposes, for each transmitted IoT datum, the times $t_{i}^{ilc}$ and $t_{i}^{olc}$ were measured, from which the delays $d_{i}^{lc}$ were then determined, according to Formula (2).

Let $t_{1}^{ilc}$ be the starting time, defined as the instant of time when the air station was directly above the ground station, $D^{lc}$ be the series of N=40,000 end-to-end delays measured at the logical channel level, starting at time $t_{1}^{ilc}$, and $D^{t}$ be the corresponding series of end-to-end delays of the same amount measured at the transport level, starting at time $t_{1}^{ilc}$. The *N* value of 40,000 provided a high delivery rate of 0.999975 for a single error. After each pair of flights, one series of delays measured at the transport level, $D_{WRTC}^{t}$, and two series of delays measured at the logical channel level, $D_{WRTC}^{lc}$ and $D_{WS}^{lc}$, were generated. The times $t_{1}^{ilc}$, at which the series $D_{WRTC}^{lc}$ and $D_{WS}^{lc}$ started, were shifted relative to each other by the time of the first flight of the pair and the service time of the second flight. The WebRTC and WS indexes indicate which web logical channel was used in a given measurement series (WebRTC data channel and WebSockets, respectively). The series $D_{WRTC}^{t}$, $D_{WRTC}^{lc}$ and $D_{WS}^{lc}$ were subjected to statistical processing.

For the analysis of the impact of single outliers, in particular the impact of the delay of IoT datum conveyed in the retransmitted packet, truncated measures were used. For this purpose, the series $D_{WRTC}^{t}$, $D_{WRTC}^{lc}$, and $D_{WS}^{lc}$ of end-to-end delays $d_{i}$, i=1,2,…,40,000 were sorted in non-decreasing order. Then, the two extreme delays (minimum and maximum delay) were discarded from the series of end-to-end delays. This resulted in new, shorter series $DT_{WRTC}^{t}$, $DT_{WRTC}^{lc}$, and $DT_{WS}^{lc}$ of end-to-end delays $d_{j}$, j=1,2,…,39,998. These series were subjected to the same statistical processing as the series from which they were derived.

### 3.3. Statistics

For time-sensitive applications, the main key performance indicator (KPI) is latency, defined as the end-to-end transmission delay. As a result, of all the related works, only Ref. [41] took latency and jitter into account, while the rest only focused on latency. Following this lead, extremes and measures of location were calculated in statistical processing. In particular, after each flight achieved

- the minimum value in each series: $min(D_{WRTC}^{t})$, $min(D_{WRTC}^{lc})$, $min(D_{WS}^{lc})$,
- the maximum value in each series: $max(D_{WRTC}^{t})$, $max(D_{WRTC}^{lc})$, $max(D_{WS}^{lc})$.

From the measures of location, both the classic measure of location, namely arithmetic mean, and the measures of position were calculated:

- arithmetic mean: $\mu (D_{WRTC}^{t})$, $\mu (D_{WRTC}^{lc})$, $\mu (D_{WS}^{lc})$:
  (3) $$\mu \left(D\right)=\frac{1}{card(D)}\sum_{i=1}^{card(D)}d_{i},d_{i}\in D$$
- median: $med(D_{WRTC}^{t})$, $med(D_{WRTC}^{lc})$, $med(D_{WS}^{lc})$,
- mode: $mod(D_{WRTC}^{t})$, $mod(D_{WRTC}^{lc})$, $mod(D_{WS}^{lc})$,
- lower quartile: $Q1(D_{WRTC}^{t})$, $Q1(D_{WRTC}^{lc})$, $Q1(D_{WS}^{lc})$,
- upper quartile: $Q3(D_{WRTC}^{t})$, $Q3(D_{WRTC}^{lc})$, $Q3(D_{WS}^{lc})$.

The same statistics, calculated from the truncated series of end-to-end delays, namely $DT_{WRTC}^{t}$, $DT_{WRTC}^{lc}$ and $DT_{WS}^{lc}$, produced truncated statistics, such as the truncated minimum $min(DT_{WRTC}^{t})$, truncated maximum $max(DT_{WRTC}^{t})$, truncated mean $\mu (DT_{WRTC}^{t})$, etc. These statistics were used to assess whether and to what extent a single retransmission affected the statistical properties of the analyzed end-to-end delays.

## 4. Experiments

Document [26] introduces a number of requirements that a WebRTC-based IoT must meet. The aim of the experiments described in this section was to check whether and to what extent the UAV-borne IoT, based on the current WebRTC standard, was able to meet the N15 requirement of [26], i.e., was able to provide low and consistent latencies under varying network conditions. As mentioned in Section 1, the challenge is in time-sensitive applications that require end-to-end delays measured in single-digit milliseconds at the application level. To meet this challenge, a WebRTC-based UAV-borne IoT should communicate with the ground station through a highly reliable, low-latency network. Since a delivery rate of 99.99% to 99.999% is considered high reliability, at most 1 packet error detected in the transport layer per 40,000 IoT data sent was assumed, i.e., a minimum packet delivery rate of 99.9975%.

The second assumption was that variable network conditions should result from both deterministic and random factors. Classic deterministic factors include the network heterogeneity (wired and wireless links), handovers, signal strength decrease with distance from access points, and UAV behavior (moving, hovering). Random factors include the different weather conditions and the different times of conducting experiments, which results in different user activities in co-existing networks in the same area, which in turn results in different loads on co-existing networks. The source of any transmission errors should be random factors.

The rest of this section presents the location of the field experiments and the course of the experiments; and discusses the flight days and sessions, network operating conditions, and the number of errors detected in the medium access control (MAC) sublayer. Finally, the measurement series selected for statistical analysis and the reasons why these series were selected and not others are described.

### 4.1. Location of the Experiments

The field experiments were carried out in a square parking lot 70 m long and 70 m wide, located on the campus of the AGH University of Krakow, Poland. The location of the experiment site between the university’s teaching buildings and the dormitory made it possible to conduct experiments at times of the day when the students’ Internet activity was low and high, generating low and high loads on the wireless networks coexisting with the air-to-ground production network in the test area. The high load on co-existing networks was a factor contributing to the occurrence of single transmission errors in the transport layer.

### 4.2. Course of Experiments

During the experiments, the air station performed automatic flights, sweeping the same 70 m × 70 m test area, zigzagging over the parking lot along the same flight path, at the same speed (1.67 m/s), and at the same altitude of 15 m. Air-to-ground transmissions were conducted both on the fly and hovering, and flight phases were intertwined with hovering phases. The summary flight time was about 460 s, and the summary hover time was about 280 s. This gave a total of just over 740 s (about 12.5 min) mission duration. The hover point locations and hover times were always the same. As the air station swept the entire test area, it switched between access points transmitting data through the 802.11ac production network described in the previous section. To ensure a seamless handover, the production network used the fast handover technique, which is part of the IEEE 802.11ac standard.

The source of the IoT data was the five environmental sensors that the air station was equipped with. During each flight, the sensors cyclically performed 9 measurements of the environmental parameters in a given time interval (0.5 s). Since each measurement datum was accompanied by two metadata (time and position), the air station sent a burst of 27 packets to the ground every half a second. This was over 1480 bursts, i.e., over 40,000 data packets, per flight. During each flight, the entry times into the logical channel, the reception times from the transport protocol (only IoT transmissions over WebRTC), and the reception times from the logical channel were collected.

During the experiments, data were sent over a web logical channel. In order to compare the IoT transmission over the WebRTC data channel with the classic solution, each evaluation flight in which IoT data were transmitted over the WebRTC data channel was followed without undue delay by a comparison flight in which IoT data were transmitted via WebSockets. This required developing a procedure for quickly replacing the web pages that included the monitoring applications, which were downloaded from a web server and run in the Chromium browser environment, which is the web server run at the WMSS. The use of a buffer power supply for both the SBC and the flight controller made it possible to change the software and replace the battery in parallel. As a result, the total elapsed time for maintenance between the evaluation flight and the next comparison flight was about 1 min.

### 4.3. Flight Days, Flight Sessions, and Pairs of Flights

The experiments were conducted from the end of January to the end of May, on separate days, on average every two weeks with an interval of at least one week, and on the same day of the week. The separation of experiments into individual days allowed the authors to run tests under different environmental conditions, such as the temperature, relative humidity, and time of day. Because the experiments started in midwinter and ended at the turn of spring to summer, transmissions were carried out from mild winter days, when the temperature rose above 1 degree Celsius, to warm late spring days, when the temperature rose to 25 degrees Celsius, and from dry weather, with a relative humidity above 40%, to rainy weather, with a relative humidity below 90%.

Experiments were organized into flight sessions. Before each session, the clocks at the air station and the ground station were synchronized. After each flight session, check-ups were performed to check for time drift, detected as a mismatch between the air and ground station clocks after the end of the session. Due to the detection of a time drift, the results collected during one flight session were rejected. Each flight session lasted up to two hours. Morning flight sessions began after 6:00 a.m. and ended before the start of classes at the University, no later than approximately 7:50 a.m. Midday flight sessions started around noon, and the evening ones started around 5 p.m. Since the experiments were conducted during the semester on campus, the time of day was related to the degree of load on the IEEE 802.11 networks coexisting with the production network on the AGH University campus and using the 5 GHz band.

The flight sessions were organized into pairs of test flights, with the evaluation flight (IoT transmission over WebRTC) immediately followed by a comparison flight (IoT transmission over WebSocket). Breaks between test flights belonging to the same pair could not be longer than would result from normal operation of the monitoring system. On a flight day, one flight session was conducted, and at least three pairs of test flights were performed during each flight session.

### 4.4. Network Conditions

Network conditions can be roughly expressed by the number of errors in the MAC sublayer: the fewer errors, the better the network conditions. The environmental conditions, especially the time of day, affected the network conditions, which were manifested in the different numbers of lost IEEE 802.11 frames per 40,000 transmitted IoT data. The number of errors in the MAC sublayer was reported by the network interface during the experiments.

Based on the number of frames lost, the network conditions were divided into good, medium, and poor. Less than two-fifths of the transmissions took place under good conditions, with just over 30 MAC frames lost per 40,000 IoT data sent. More than two-fifths were carried out under medium conditions, with more than 40 and no more than about 95 frames lost. More than one fifth of the transmissions took place under poor network conditions, when approximately 100 frames or more were lost per 40,000 IoT data transmitted.

The IEEE 802.11ac error control mechanism successfully retransmitted almost all lost frames detected by the MAC sublayer. Under both good and medium network conditions, the MAC sublayer was always able to correct the transmission errors. As an effect, no errors were detected at the transport layer. In poor network conditions, the underlying network was always unable to successfully retransmit one lost frame. As a result, a single transmission error (one lost packet per 40,000 IoT data sent) was detected at the transport layer. Errors in the transport layer usually appeared during both flights from a given pair. There was only one registered exception to this rule, when the transmission of IoT data over the WebRTC data channel was error-free at the transport layer, while during the transmission of IoT data over the WebSocket, the TCP detected a single transmission error.

### 4.5. Selection of Measurement Series

Out of 35 pairs of flights, we selected five, conducted on five different flight days, when transmissions were carried out under the three different network conditions (good, medium, poor):

- On day 1, transmissions were carried out in good network conditions. No errors were detected in the transport layer for both IoT data transmission over the WebRTC Data Channel and over the WebSocket. Thus, the packet error rate (PER) in the transport layer was $PER_{WRTC}=PER_{WS}=0$.
- On day 2, network conditions were on the border between medium and poor. During the first flight, the exception described in previous section occurred: no errors were detected in the transport layer when transmitting IoT data over the WebRTC data channel, and one error was detected during transmission over WebSocket. $PER_{WRTC}$ was 0 and $PER_{WS}$ was 0.0025%.
- On day 3 transmissions were again carried out under good network conditions. No errors were detected in the transport layer during both transmissions ($PER_{WRTC}=PER_{WS}=0$).
- On the fourth day, transmissions took place under poor network conditions. Each transport protocol detected one transmission error ($PER_{WRTC}=PER_{WS}=0.0025\%$).
- On day 5, transmissions were conducted under medium network conditions. No errors were detected in the transport layer during both transmissions ($PER_{WRTC}=PER_{WS}=0$).

The five selected pairs of flights were conducted during different flight sessions (a morning session, a midday session, and an evening session) and under different weather conditions: from cold days to warm days (1.5 to 25 degrees Celsius), during dry, wet, and just after rainy weather (relative humidities from 46% up to 85%).

## 5. Results

This section presents and discusses the results of the field experiments, to verify whether a UAV and WebRTC-based IoT is suitable for time-sensitive applications operating in a highly mobile outdoor environment when the underlying network is capable of providing a reliable, low-latency communication service.

### 5.1. WebRTC Data Channel: Minimum and Maximum of the End-to-End Delays Measured at the Transport Level

Table 3 includes the minimum $min(D_{WRTC}^{t})$ and maximum $max(D_{WRTC}^{t})$ of the end-to-end delays measured at the transport level when the IoT data were transmitted over the WebRTC data channel, and the truncated maximum $max(DT_{WRTC}^{t})$, calculated after discarding the extreme values from the series of end-to-end delays $D_{WRTC}^{t}$. Day 4 was the only day on which the PER was not equal to zero, and the large maximum delay recorded on that day was the retransmitted packet delay.

During the IoT transmissions over the WebRTC data channel carried out on day 1 to day 3 and day 5, where no transmission errors were detected at the transport layer, the maximum end-to-end delay measured at the transport level always achieved a single-digit millisecond value (Table 3). Each of the four transmissions achieved a maximum end-to-end delay of 3.5 ms (3469 µs). The maxima of the truncated series were also 3469 µs. This extremely high repeatability of the values of maxima and truncated maxima obtained on different days, when the values were repeated with an accuracy of one microsecond, may indicate an exceptionally high stability of the transmissions conducted under good and average network conditions.

The end-to-end delay minima did not show such outstanding stability, in the sense of the repeatability of results, over the different experiments carried out in the lossless environment. But even here, when the PER was zero, the differences between the results obtained on the different days did not exceeded 30 µs (values from 3377 µs to 3404 µs), which is less than 1% of the minimum values. As an effect, compared to the extremes calculated for the transmission error experiment conducted on day 4, both the maximum and the minimum can be considered stable across the error-free experiments carried out on the same network, but under different network conditions.

**Figure 2:**
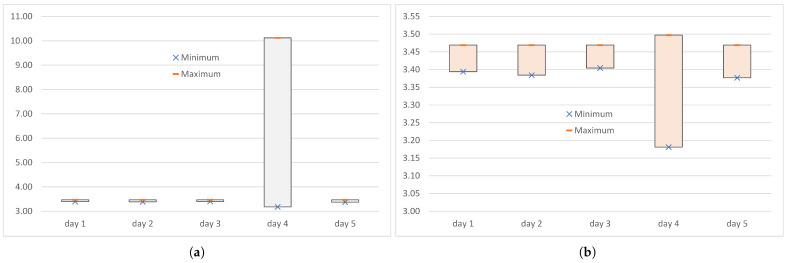
The range of end-to-end delays (in ms) measured at the transport level when the IoT data were transmitted over the WebRTC data channel: (**a**) full series $D_{WRTC}^{t}$; (**b**) truncated series $DT_{WRTC}^{t}$.

The single transmission error that occurred in the experiment conducted on day 4 affected both the maximum value of end-to-end delay measured at the transport level and the minimum value. Because the transmission error led to the retransmission of the lost packet, the maximum end-to-end delay was 10,120 µs, which is more than three times higher than the maxima obtained during the error-free transmissions (Figure 2a). When the delay of the retransmitted packet was discarded from the series of end-to-end delays, the maximum value dropped to 3497 µs. This is less than 1% above the maximum obtained during the error-free transmissions (Figure 2b). This shows that, at the transport level, this large increase in delay was local and its impact was limited to a single error correction via selective retransmission. The SCTP packets, except the retransmitted packet, were transmitted with delays suitable for time-sensitive applications.

The occurrence of an error not only increased the maximum, but also lowered the minimum (Figure 2). For day 4, the minimum was 3181 µs, which is about 10% (300 µs) less than on the other days. Analysis of the instantaneous values (in some publications, e.g., Ref. [37], also called real-time values) of the end-to-end delay shows that the delay of the next burst after the packet loss decreased, then started to increase, and after a few seconds returned to the level observed before the transmission error. The truncated minimum, calculated after discarding the minimum and the maximum delay from the series of delays, was the same as the minimum (i.e., 3181 µs).

### 5.2. WebRTC Data Channel: Minimum and Maximum of the End-to-End Delays Measured at the Logical Channel Level

While delays measured at the transport level refer to the moment at which the MQTT message decapsulated from the SCTP packet is placed in the receive buffer of the logical channel, delays measured at the logical channel level refer to the moment at which the MQTT is informed that the MQTT message is ready for reception. Table 4 includes the minimum $min(D_{WRTC}^{lc})$, maximum $max(D_{WRTC}^{lc})$, and truncated maximum $max(DT_{WRTC}^{lc})$ of the end-to-end delays measured at the logical channel level when the IoT data embedded in MQTT messages were transmitted over the WebRTC data channel.

The difference of two microseconds between almost all of the statistics listed in Table 3, except the truncated maximum, and the corresponding statistics listed in Table 4 is the processing time of WebRTC data channel that processed the payload of the SCTP packet buffered in the receive buffer.

**Figure 3:**
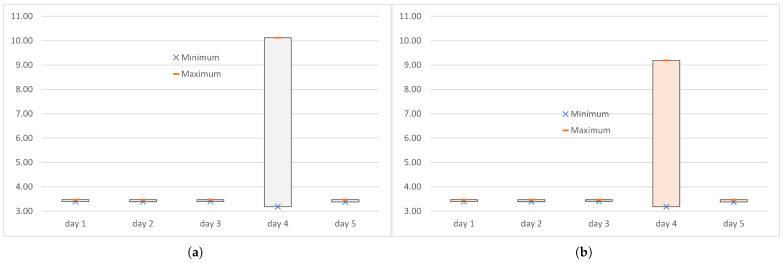
The range of end-to-end delays (in ms) measured at the logical channel level when the IoT data were transmitted over the WebRTC data channel: (**a**) full series $D_{WRTC}^{lc}$; (**b**) truncated series $DT_{WRTC}^{lc}$.

A large difference is visible on day 4 between the maxima of the truncated end-to-end delay series measured at the transport level and at the logical channel level. While, at the transport level, the truncated maximum was about 3.5 ms (precisely: 3497 µs), at the logical channel level, it was about 9 ms (9187 µs). The truncated maximum calculated at the logical channel level (Figure 3b) was more than two and a half times larger than the truncated maximum obtained at the transport level (Figure 2b), and only about 10% less than the end-to-end delay of the retransmitted packet (10,122 µs), as presented in Figure 3a. Such a large difference resulted from the fact that the packets already received, but sent after the lost packet, were waiting for the retransmission of the lost packet. Only when the retransmitted packet was transferred to the receive buffer of the logical channel did the MQTT protocol receive information that these packets were ready.

### 5.3. WebSocket: Minimum and Maximum of End-to-End Delays Measured at the Logical Channel Level

Table 5 lists the minimum $min(D_{WS}^{lc})$, maximum $max(D_{WS}^{lc})$, and the truncated maximum $max(DT_{WS}^{lc})$ values of the end-to-end delays measured at the logical channel level when the MQTT messages were transmitted over the WebSocket. The end-to-end delays of the transmission of IoT data over the WebSocket, measured during experiments conducted on days 1 to 5, had relatively small minima (2515 µs to 2550 µs), smaller than minima of the delays of transmissions over WebRTC (Table 4). However, the maxima were very large, at 65,723 µs to 87,145 µs.

**Figure 4:**
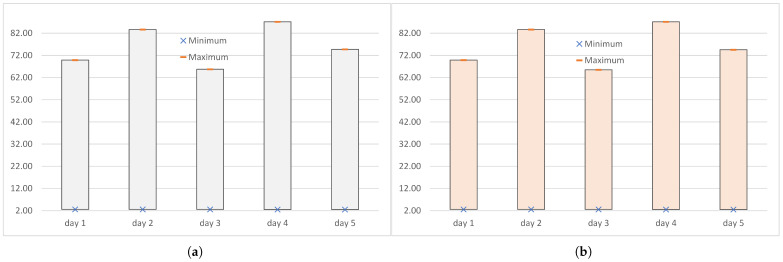
The range of end-to-end delays (in ms) measured at the logical channel level when the IoT data were transmitted over the WebSocket: (**a**) full series $D_{WS}^{lc}$; (**b**) truncated series $DT_{WS}^{lc}$.

The occurrence of packet loss on days 2 and 4 slightly (by 20–30 µs) lowered the minimum and significantly (by 10–20 ms) increased the maximum compared to the experiments in which PER was equal to 0. Unlike the end-to-end delays measured during transmissions over WebRTC (Figure 3), discarding the extreme delays from the series of delays did not lower the maximum value enough to be practical (Figure 4). When the PER was non-zero, the truncated maximum was 2 µs (day 4) to 13 µs (day 2) less than the maximum, and when the PER was zero, the truncated maximum was 11 µs (day 1) to 234 µs (day 3).

### 5.4. WebRTC: Measures of Location

Table 6 and Table 7 present the mean $\mu (D_{WRTC}^{t})$ and $\mu (D_{WRTC}^{lc})$, median $med(D_{WRTC}^{t})$ and $med(D_{WRTC}^{lc})$, mode $mod(D_{WRTC}^{t})$ and $mod(D_{WRTC}^{lc})$, upper quartile $Q3(D_{WRTC}^{t})$ and $Q3(D_{WRTC}^{lc})$, and lower quartile $Q1(D_{WRTC}^{t})$ and $Q1(D_{WRTC}^{lc})$ of the end-to-end delays measured at the transport level and at the logical channel level, respectively, when the MQTT messages were transmitted over the WebRTC data channel. The results in Table 6 and Table 7 differ by 2 microseconds. This was a delay resulting from the processing in the receive buffer of the logical channel.

Because for days 1–3 and day 5, when PER=0, the maxima of end-to-end delays were less than 10 ms, the measures of location for these days were also less than 10 ms (Table 6 and Table 7). Because the non-zero PER that occurred on day 4 was small (one packet lost per 40,000 packets sent), a single outlier (transport level) or a small group of outliers (logical channel level) were unable to influence either the arithmetic mean or measures of position. As an effect, all measures of location presented in Table 6 and Table 7 are one-digit milliseconds.

In the case of error-free transmission in the transport layer (PER equal to 0), which was carried out on day 1 to 3 and day 5, the measures of location calculated both at the transport level and at the logical channel level showed similar extremely high repeatability as the maximums of error-free transmissions shown in the previous section. At the logical channel level, the arithmetic mean of the end-to-end delays was 3461 to 3462 µs (i.e., 3461.5 ± 0.5 µs), the median was 3468 to 3469 µs (i.e., 3468.5 ± 0.5 µs), the mode was 3470 µs and equaled the upper quartile, and the lower quartile was 3456 to 3460 µs (i.e., 3458 ± 2 µs). At the transport level, the measures of location were reduced by 2 µs, and the abovementioned numerical relationships between the statistical measures were the same.

**Figure 5:**
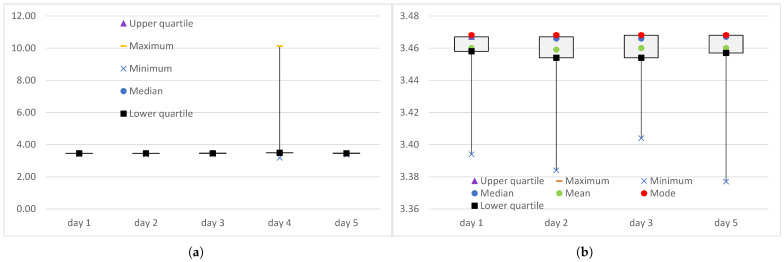
The five-number summary of the end-to-end delays (in ms) measured at the logical channel level when the IoT data were transmitted over WebRTC: (**a**) all experiments (PER>=0); (**b**) the transport layer considered the transmission to be error-free (PER=0). For PER=0, the arithmetic mean and the mode are also shown.

The stability of the measures of location over the different experiments, observed for PER=0, was accompanied by very small differences between the measures (Figure 5b). The upper quartile was 1–2 microseconds greater than the median, and the lower quartile was about 10 ms lower than the median. The differences between the maximums and medians were also of a few microseconds. At the logical channel level, the maximum end-to-end delay was 3471 µs, while the median of these delays was 2 to 3 µs smaller. Because the above numerical relationships were preserved at the transport level, at least 50% of the end-to-end delays were maximum at both considered levels (within 3 microseconds). This indicated a strong stability for the transmissions performed on days 1–3 and day 5, with a very small jitter.

The poor network condition on day 4 caused a single transmission error that was detected by the transport protocol, and then the lost packet was retransmitted. The end-to-end delays measured on this day had higher values for all measures of location, both the mean and quartiles, by about 30 µs in relation to the measures calculated for error-free transmissions. Due to this uniform shift, the numerical relationships between measures of position were the same as observed in the case of error-free transmissions: the mode was equal to the upper quartile, the upper quartile was 1–2 microseconds (here, 1 µs) greater than the median, and the lower quartile was about 10 microseconds (here, 9 µs) below the median (Table 6 and Table 7). Since the maximum end-to-end delay was the delay of the IoT datum sent in the retransmitted packet, the difference between the median and the maximum value was more than 6.5 ms (Figure 5a). The values of the maximum and the median end-to-end delay cannot therefore be considered close. However, in the case of delays measured at the transport level, the truncated median (3495 µs) and the truncated maximum (3497 µs) were close to each other, differing by only 2 µs. In the case of delays measured at the logical channel level, due to the long waiting time for packets to be sorted out in the receive buffer after retransmitting a lost packet, the truncated median (3.497 µs) and the truncated maximum (9.187 µs) differed by 5.690 µs.

### 5.5. WebSockets: Measures of Location

**Figure 6:**
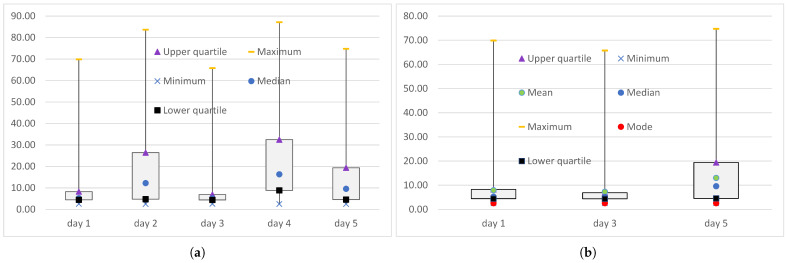
The five-number summary of end-to-end delays (in ms) measured at the logical channel level when IoT data were transmitted over WebSocket: (**a**) all experiments (PER>=0); (**b**) the transport layer considered the transmission to be error-free (PER=0). For PER=0 the arithmetic mean and the mode are also shown.

Table 8 summarizes the measures of location: mean $\mu (D_{WS}^{lc})$, median $med(D_{WS}^{lc})$, mode $mod(D_{WS}^{lc})$, upper quartile $Q3(D_{WS}^{lc})$, and lower quartile $Q1(D_{WS}^{lc})$ of the end-to-end delays measured at the logical channel level when MQTT transmissions were carried out over the WebSocket. While all measures of the location of end-to-end delays of IoT data transmitted over WebRTC satisfied the single-digit millisecond requirements of time-sensitive applications (Table 7, Figure 5a), the lower quartile and the mode were the only measures of location that always met this requirement when transmissions were carried out over the classic web logical channel (Table 8, Figure 6a). The median only met this requirement on days 1, 3, and 5, when the underlying IEEE 802.11 network was able to ensure reliable transmission in the transport layer. The arithmetic mean and upper quartile only met it when the error rate in the MAC sublayer was not greater than 0.1 percent (day 1 and day 2).

The comparison of the results presented in Table 7 and Table 8 shows that the mode was the only measure of location in terms of which the IoT transmissions over WebSocket were superior to IoT transmissions over WebRTC Data Channel. Two such measures were therefore found: the mode and minimum (see previous sections). In IoT transmissions using WebSocket, the mode was not equal to the upper quartile, as in the IoT transmissions using WebRTC (Figure 5b), but to the minimum (Figure 6b). However, the relatively small number of delays whose value was a modal value (WebRTC: 12–15 thousand on days 1–3 and 5, over 10 thousand on day 4; WebSocket: about 1 thousand on days 1, 2, and 5, about 500 on day 2, no modal value on day 4) shows that this modal superiority of transmissions over WebSocket does not matter much in practice.

## 6. Discussion

The previous section presented and analyzed the minimum, maximum, arithmetic mean, mode, and quartiles (upper, median, and lower quartile) of the end-to-end delays of air-to-ground IoT transmissions carried out using the WebRTC data channel or WebSocket. Table 9 contains a comparison of the statistics collected at the logical channel level, in the form of the ratio of the value of a given statistical measure calculated for IoT transmission via WebSocket (Table 5 and Table 8) to the value of the same measure calculated for IoT transmission via WebRTC (Table 4 and Table 7). If the values of the same statistical measure calculated for transmissions using WebRTC Data Channel and WebSocket are equal, the ratio will be 1. In such a situation, it will not matter, from the point of view of a given statistical measure, through which of the web logical channels the IoT transmission is carried out between the UAV and the ground. This is a purely hypothetical case and does not appear in Table 9.

A ratio of statistical measures less than 1 indicates the superiority of the WebSocket web logical channel over the WebRTC data channel. This ratio appears in Table 9 twice: for minimum values and for modal values. The ratio of minima, $min(D_{WS}^{lc})$ to $min(D_{WRTC}^{lc})$, ranged from 0.74 for day 2 to 0.79 for day 4. This means that, when using WebRTC, the minimum end-to-end delays were approximately one third greater under good and medium network conditions and approximately a quarter greater under poor network conditions than the minimum delays achieved when transmitting using WebSockets. The ratio of modal values, $mod(D_{WS}^{lc})$ to $mod(D_{WRTC}^{lc})$, amounted to 0.73–0.74 for IoT transmissions under good and medium network conditions. Under the poor network conditions, no modal value was observed during transmission using WebSockets. However, the relatively small number of minimally delayed packets made this advantage of WebSockets over WebRTC relatively minor.

**Figure 7:**
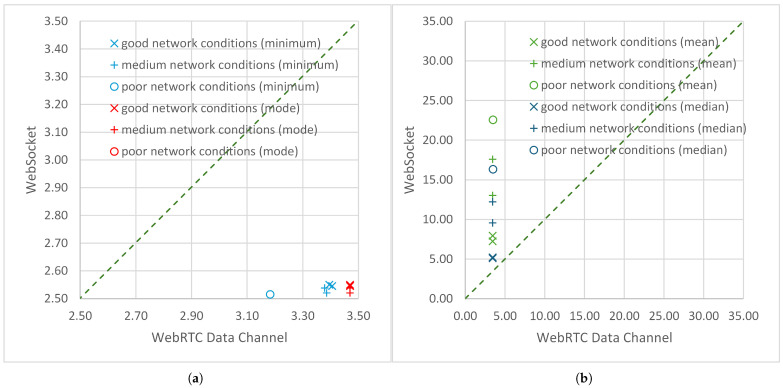
Scatter plots for the statistics of the end-to-end delays (in ms) measured at the logical channel level during air-to-ground transmissions using the WebRTC data channel and using the WebSocket logical channel: (**a**) minimum and mode; (**b**) mean and median.

**Figure 8:**
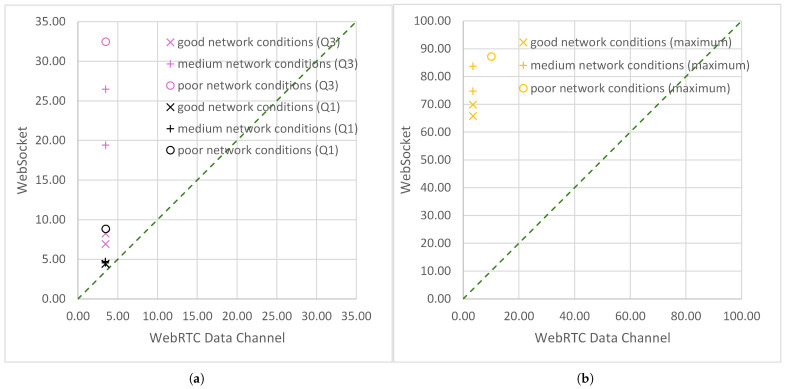
Scatter plots for statistics of end-to-end delays (in ms) measured at the logical channel level during air-to-ground transmissions using WebRTC data channel and using the WebSocket logical channel: (**a**) lower quartile and upper quartile; (**b**) maximum.

Figure 7 and Figure 8 show scatter plots drawn for the end-to-end delay statistics calculated at the logical channel level and presented in the previous section. The values obtained for transmissions using WebSockets (Table 5 and Table 8) are plotted as a function of the corresponding values obtained for the transmissions using WebRTC (Table 4 and Table 7). The markers denote statistics calculated for transmissions under good (x), average (+), and poor (o) network conditions. The diagonal of each plot (dashed line) illustrates the hypothetical case of a ratio of a given statistical measure equal to 1. Below the diagonal, there were only minimum and mode markers (Figure 7a). The values of these statistics for transmissions using WebRTC were higher than for transmissions using WebSockets, so the ratio given in Table 9 is less than 1. The highest minimum latencies and highest latency modes were observed under good network conditions. As the network conditions deteriorated, the minimum and mode began to decrease, although the observed differences were small, and when using WebRTC, there was no difference between the modes calculated under good and medium network conditions. The lowest minimum delay occurred under poor network conditions, but only in the case of WebRTC was the reduction in the minimum significant.

A ratio of statistical measures greater than 1 indicates the superiority of the WebRTC data channel over WebSocket. This ratio appears in Table 9 for the arithmetic mean, quartiles, and maximum value. Except for the latter, unlike the case of ratios smaller than 1, the least ratios greater than 1 were obtained under good network conditions, and as the network conditions deteriorated, the ratio value increased. As an example, the ratio of arithmetic means, $\mu (D_{WS}^{lc})$ to $\mu (D_{WRTC}^{lc})$, ranged from 2.1 for day 3 to 6.47 for day 4. Under good network conditions, the mean delay of air-to-ground IoT transmissions using WebSockets was more than twice the mean delay of transmissions using WebRTC. Under medium network conditions, it was almost four times greater. When the network conditions were on the verge of medium to poor, the mean delay of transmissions using WebSocket was just over five times greater, and when the network conditions were poor, it was well over six times greater than the mean delay of transmissions using WebRTC (Table 9, Figure 7b). In addition, in the case of the ratio of quartiles, the greater the distance (in the number of samples, or position) of a given measure from the minimum, the greater the ratio values. The ratio of lower quartiles, $Q1(D_{WS}^{lc})$ to $Q1(D_{WRTC}^{lc})$, ranged from 1.27 for day 3 to 2.53 for day 4 (Table 9, Figure 8a). The ratio of medians, $med(D_{WS}^{lc})$ to $med(D_{WRTC}^{lc})$ ranged from 1.48 for day 3 to 4.67 for day 4 (Table 9, Figure 7b). The ratio of upper quartiles, $Q3(D_{WS}^{lc})$ to $Q3(D_{WRTC}^{lc})$, ranged from 1.27 for day 3 to 9.28 for day 4 (Table 9, Figure 8a).

In the case of end-to-end delay maxima ratios, $max(D_{WS}^{lc})$ to $max(D_{WRTC}^{lc})$, the above observations were true for days 1 to 3 and day 5, when transmission at the transport layer was error-free. Under good network conditions, the maximum delay of transmission using WebSocket was approximately 20 times greater than the maximum delay of transmission using WebRTC. When the network conditions were medium, it was about 22 times greater, and when the network conditions were between medium and poor, it was more than 24 times greater than the maximum delay of transmission using WebRTC. In the case of a single transmission error (day 4), the ratio of maxima dropped to over 8 (Table 9), due to the large increase in the maximum delay in transmission using WebRTC caused by packet retransmission. As with all other measures for which the ratio was greater than 1 (Figure 7b and Figure 8a), the maximum transmission delay using the WebSocket was the highest under poor network conditions (Figure 8b).

When comparing the obtained results with those reported in related works, the delay introduced by the implementation of the MQTT protocol should be taken into account. Additional laboratory experiments showed that the classic Eclipse Paho JavaScript Client implementation of the MQTT protocol introduced delays averaging approximately 1.5 ms. For WebSocket-based IoT, this resulted in average application-level end-to-end delays of approximately 9 ms to 24 ms. Taking into account that similar delays during transmission from the UAV to the ground station in systems using WebSocket have been reported in the literature (e.g., 23 ms [30], 20 ms to 25 ms in the IEEE 802.11 network [34]), it can be concluded that that the end-to-end delay values for UAV-borne IoT using WebSocket were comparable to those reported in related works.

In the case of solutions other than WebSocket, but still based on the TCP protocol, the situation is similar. In [32], replacing single-path transmission using TCP with a multi-path transmission using the improved MPTCP reduced the latency from 920.4 ms to 568.1 ms (i.e., 1.62 times). This was achieved at the expense of the parallel transmission of cloned packets. However, in this paper, the use of WebRTC provided a greater relative improvement than [32] and without as much computational and energy cost. Significantly, modifying the MPTCP so that the UDP was used as the underlying protocol instead of the TCP [31] allowed for a relative improvement similar to that shown in this paper, and at lower computational costs than in [32] due to the simplicity of the UDP mechanisms. However, the energy cost of the solution proposed in [31] remained significant. Moreover, since the STCP implements multihoming, multipath transmissions of cloned sensor data can also be used in WebRTC-based IoT.

It can be expected that the advantages of the WebRTC data channel used in UAV communication may be comparable to those of using any other UDP-based solution. Comparing the results of experiments on MQTT over WebRTC data channel presented in this work with the results of the experiments on MQTT over QUIC presented in [38], it can be seen that, in the case of error-free transmissions, the results were approximately similar. If the latencies introduced by the underlying IEEE 802.11 networks (2 ms in this article and 25 ms in [38]), as well as the estimated delays introduced by the Paho implementation of the MQTT protocol (1.5 ms), are subtracted from the average results, the approximate average delays obtained in this paper and in [38] are the same and equal 1.5 ms. However, the spread of end-to-end delay values was much larger in [38] than in this work. Due to the significant differences in the test environment (in this paper, a mobile and highly variable real-world environment using a low-latency network was employed; a static environment using an emulated high-latency network was employed in [38]), it is impossible to say with certainty how beneficial it would be to use a WebRTC data channel in UAV-IoT communication instead of QUIC.

The second example of a UDP-based solution is presented in [37], which compared IoT transmissions over SCTP with IoT transmissions over TCP. The results of simulation experiments showed the better performance of SCTP and better stability of TCP in heterogeneous networks (wired and wireless). During error-free transmissions, the performance difference between SCTP and TCP shown in [37] was not as large as the performance difference estimated from the results presented in this paper. The issue of stability was also different: in this paper, the SCTP was extremely stable, and much more stable than the TCP. It is worth emphasizing here that, in [37], an older version of the SCTP was discussed, and the research conducted in this paper used a new, WebRTC-oriented version of the SCTP that is currently implemented in web browsers.

## 7. Conclusions

In recent years, wireless communication for time-sensitive IoT applications has become a hot research topic, including applications that require end-to-end delays measured in single-digit milliseconds. One of the problems encountered in these applications is the processing in higher network layers: even if the underlying network is capable of providing highly reliable, low-latency communications, the delays introduced at the transport layer and above may prove too great to meet stringent time requirements. The aim of this paper was to show that, in the case of IoT carried by UAV, the use of WebRTC can help solve this problem.

The paper used high-resolution time measurement procedures and timer synchronization to perform delay measurements at the level of the transport protocol and at the level of the network logical channel of the WebRTC IoT application, run on board the UAV. During the field experiments, air-to-ground IoT transmissions were carried out under various network conditions, followed by statistical analysis of these delays, focusing on extreme values and location measures. The obtained results were compared with those obtained for IoT transmission via the WebSocket logical channel, under the same circumstances.

The statistical characteristics of the end-to-end delays showed that, during air-to-ground transmission, the WebRTC-based IoT was able to achieve single-digit-millisecond end-to-end delays on both the transport protocol level and the logical channel level. When the WebRTC transmission was error-free, stable end-to-end delays well below 10 ms were achieved. When a single transmission error occurred, higher end-to-end delays were observed in the immediate vicinity of the retransmitted packet, although they were still below 10 ms. Only the delay of the retransmitted packet slightly exceeded 10 ms.

The results of the same IoT transmissions performed via WebSocket under the same circumstances showed that the WebRTC-based UAV-borne IoT had 8.5 to 24 times lower maximum delays and 2 to 6.5 times lower mean delays than the same IoT using WebSocket. The smallest differences between the maximum values and the largest differences between the arithmetic means were associated with the occurrence of a transmission error. The results therefore indicated the superiority of the WebRTC logical channel over the classic web logical channel.

Future research will focus on analyzing WebRTC-based UAV-borne IoT transmissions in Wi-Fi 6e and Wi-Fi 7 networks, as well as UAV swarm tests in a 5G test network.

## Figures and Tables

**Table 1:** Selected time-sensitive applications with stringent time constraints related to both UAVs and IoT that can be carried by UAVs.

Application	Time Constraint	Paper
Connecting autonomous vehicles	below 1 ms	[21]
Transport industry	3 or 7 ms	[22]
Intelligent transportation system	5 to 10 ms	[23]
Internet of drones (remote control)	5 to 50 ms	[23]
Approaching autonomous navigation infrastructure	10 ms	[24]
Mobile robots: video-operated remote control	10 to 100 ms	[25]
Command and control of UAV networks	10, 40, or 140 ms	[22]

**Table 2:** Related work.

Paper	Carrier	Network Technology	Transport Protocol	Web Logical Channel	API
[15]	UAV	5G	n/a ^1,5^	n/a ^1,5^	n/a ^1,5^
[27,28]	UAV	802.11ac	RTP, SCTP	WebRTC	WebRTC
[30]	UAV	5G, 802.11p	TCP ^4^	WebSocket	n/a ^1,5^
[31,32]	UAV	802.11g	MPTCP	n/a ^1^	socket
[33]	UAV	802.11n	MPTCP	n/a ^1^	socket
[34,35]	UAV	802.11n	RTP ^4^, TCP ^4^	WebRTC, WebSocket	WebRTC, WebSocket
[36]	UAV	802.11	TCP ^4^	WebSocket	WebSocket
[41]	UAV	4G	UDP	n/a ^1^	n/a ^1,5^
			TCP	n/a^1^	n/a ^1,5^
[44]	n/a ^1,3^, UGV ^3^	802.11	TCP ^4^	WebSocket	WebSocket
			RTP ^4^	WebRTC	WebRTC
[45]	UGV	SDR	TCP ^4^	WebSocket	WebSocket
[46]	n/a ^1,2^	802.11, 3.75G	TCP ^4^, RTP ^4^, SCTP ^4^	WebSocket, WebRTC	WebSocket, WebRTC
[38,39]	n/a ^1^	802.11, Cellular	QUIC, TCP	n/a ^1,5^	socket
		Satellite			
[37]	n/a ^1^	802.11	SCTP, TCP	n/a ^1,5^	n/a ^1,5^
[40]	UAV	802.11n, 4G, 5G	SCTP	WebRTC	WebRTC
this paper	UAV	802.11ac	SCTP	WebRTC	WebRTC
			TCP	WebSocket	WebSocket

^1^ not applicable, ^2^ stationary robot, ^3^ the target application is UAV, ^4^ stated implicitly, ^5^ simulation.

**Table 3:** Minimum and maximum of the end-to-end delays measured at the transport level when the IoT data were transmitted over the WebRTC data channel.

Days	$\min(D_{\mathrm{WRTC}}^{t})$	$\max(D_{\mathrm{WRTC}}^{t})$	$\max({\mathrm{DT}}_{\mathrm{WRTC}}^{t})$ ^1^
day 1	3394 µs	3469 µs	3469 µs
day 2	3384 µs	3469 µs	3469 µs
day 3	3404 µs	3469 µs	3469 µs
day 4	3181 µs	10,120 µs	3497 µs
day 5	3377 µs	3469 µs	3469 µs

^1^ Maximum of the series of end-to-end delays truncated by the extremes.

**Table 4:** Minimum, maximum, and truncated maximum of end-to-end delays measured at the logical channel level when the IoT data were transmitted through the WebRTC data channel.

Days	$\min(D_{\mathrm{WRTC}}^{\mathrm{lc}})$	$\max(D_{\mathrm{WRTC}}^{\mathrm{lc}})$	$\max({\mathrm{DT}}_{\mathrm{WRTC}}^{\mathrm{lc}})$ ^1^
day 1	3396 µs	3471 µs	3471 µs
day 2	3386 µs	3471 µs	3471 µs
day 3	3406 µs	3471 µs	3471 µs
day 4	3183 µs	10,122 µs	9187 µs
day 5	3379 µs	3471 µs	3471 µs

^1^ Maximum of the series of end-to-end delays truncated by the extremes.

**Table 5:** Minimum and maximum of the end-to-end delays measured at the logical channel level when the IoT data were transmitted through the WebSocket logical channel.

Days	$\min(D_{\mathrm{WS}}^{\mathrm{lc}})$	$\max(D_{\mathrm{WS}}^{\mathrm{lc}})$	$\max({\mathrm{DT}}_{\mathrm{WS}}^{\mathrm{lc}})$ ^1^
day 1	2550 µs	69,865 µs	69,854 µs
day 2	2520 µs	83,652 µs	83,649 µs
day 3	2545 µs	65,723 µs	65,489 µs
day 4	2515 µs	87,145 µs	87,143 µs
day 5	2538 µs	74,722 µs	74,549 µs

^1^ Maximum of the series of end-to-end delays truncated by the extremes.

**Table 6:** Measures of location (mean, median, mode, upper quartile, and lower quartile) of end-to-end delays measured at the transport level when the IoT data were transmitted through the WebRTC data channel.

Days	$\mu (D_{\mathrm{WRTC}}^{t})$	$\mathrm{med}(D_{\mathrm{WRTC}}^{t})$	$\mathrm{mod}(D_{\mathrm{WRTC}}^{t})$	$Q3(D_{\mathrm{WRTC}}^{t})$	$Q1(D_{\mathrm{WRTC}}^{t})$
day 1	3460 µs	3467 µs	3468 µs	3468 µs	3458 µs
day 2	3459 µs	3466 µs	3468 µs	3468 µs	3454 µs
day 3	3460 µs	3466 µs	3468 µs	3468 µs	3455 µs
day 4	3492 µs	3495 µs	3496 µs	3496 µs	3486 µs
day 5	3460 µs	3467 µs	3468 µs	3468 µs	3457 µs

**Table 7:** Measures of location of end-to-end delays measured at the logical channel level when the IoT data were transmitted over the WebRTC data channel.

Days	$\mu (D_{\mathrm{WRTC}}^{\mathrm{lc}})$	$\mathrm{med}(D_{\mathrm{WRTC}}^{\mathrm{lc}})$	$\mathrm{mod}(D_{\mathrm{WRTC}}^{\mathrm{lc}})$	$Q3(D_{\mathrm{WRTC}}^{\mathrm{lc}})$	$Q1(D_{\mathrm{WRTC}}^{\mathrm{lc}})$
day 1	3462 µs	3469 µs	3470 µs	3470 µs	3460 µs
day 2	3461 µs	3468 µs	3470 µs	3470 µs	3456 µs
day 3	3462 µs	3468 µs	3470 µs	3470 µs	3457 µs
day 4	3494 µs	3497 µs	3498 µs	3498 µs	3488 µs
day 5	3462 µs	3469 µs	3470 µs	3470 µs	3459 µs

**Table 8:** Measures of location (mean, median, mode, upper quartile, and lower quartile) of end-to-end delays measured at the logical channel level when the IoT data were transmitted over the WebSocket.

Days	$\mu (D_{\mathrm{WS}}^{\mathrm{lc}})$	$\mathrm{med}(D_{\mathrm{WS}}^{\mathrm{lc}})$	$\mathrm{mod}(D_{\mathrm{WS}}^{\mathrm{lc}})$	$Q3(D_{\mathrm{WS}}^{\mathrm{lc}})$	$Q1(D_{\mathrm{WS}}^{\mathrm{lc}})$
day 1	7947 µs	5235 µs	2550 µs	8247 µs	4431 µs
day 2	17,605 µs	12,233 µs	2520 µs	26,445 µs	4721 µs
day 3	7260 µs	5123 µs	2545 µs	6919 µs	4403 µs
day 4	22,584 µs	16335 µs	-	32,448 µs	8833 µs
day 5	13,030 µs	9563 µs	2538 µs	19,389 µs	4537 µs

**Table 9:** Comparison of extremes (minimum and maximum) and measures of location (mean, median, mode, upper quartile, and lower quartile) of the end-to-end delays measured at the logical channel level when the IoT data were transmitted using WebRTC data channel and WebSocket.

Days	$\frac{\min(D_{\mathrm{WS}}^{\mathrm{lc}})}{\min(D_{\mathrm{WRTC}}^{\mathrm{lc}})}$	$\frac{\max(D_{\mathrm{WS}}^{\mathrm{lc}})}{\max(D_{\mathrm{WRTC}}^{\mathrm{lc}})}$	$\frac{\mu (D_{\mathrm{WS}}^{\mathrm{lc}})}{\mu (D_{\mathrm{WRTC}}^{\mathrm{lc}})}$	$\frac{\mathrm{med}(D_{\mathrm{WS}}^{\mathrm{lc}})}{\mathrm{med}(D_{\mathrm{WRTC}}^{\mathrm{lc}})}$	$\frac{\mathrm{mod}(D_{\mathrm{WS}}^{\mathrm{lc}})}{\mathrm{mod}(D_{\mathrm{WRTC}}^{\mathrm{lc}})}$	$\frac{Q3(D_{\mathrm{WS}}^{\mathrm{lc}})}{Q3(D_{\mathrm{WRTC}}^{\mathrm{lc}})}$	$\frac{Q1(D_{\mathrm{WS}}^{\mathrm{lc}})}{Q1(D_{\mathrm{WRTC}}^{\mathrm{lc}})}$
day 1	0.75	20.14	2.3	1.51	0.74	2.38	1.28
day 2	0.74	24.11	5.09	3.53	0.73	7.62	1.37
day 3	0.75	18.95	2.1	1.48	0.73	1.99	1.27
day 4	0.79	8.61	6.47	4.67	-	9.28	2.53
day 5	0.75	21.54	3.77	2.76	0.73	5.59	1.31

## Data Availability

The data supporting the conclusions of this article will be made available by the authors on request.

## Abbreviations

The following abbreviations are used in this manuscript:

**Abbreviations**	
4G	Fourth-generation (technology for broadband cellular networks)
5G	Fifth-generation (technology for broadband cellular networks)
AMEC	Aerial mobile edge computing
AMQP	Advanced Message Queuing Protocol
API	Application programming interface
CPU	Central processing unit
CCC	Command and control console
DiffServ	Differentiated Services
ESS	Extended Service Set
HSPA+	Evolved High Speed Packet Access (Evolved HSPA, HSPA Evolution)
HTTP	Hypertext Transfer Protocol
IEEE	Institute of Electrical and Electronics Engineers
IoT	Internet of Things
ISP	Internet service provider
KPI	Key performance indicator
LRT	Low-latency Reliable Transmission
LTE	Long Term Evolution
MAC	Medium access control
MPTCP	Multipath Transmission Control Protocol (Multipath TCP)
MQTT	Message Queuing Telemetry Transport (MQ Telemetry Transport)
PAV	Personal air vehicle
PER	Packet error rate
QoS	Quality of Service
QUIC	Quick UDP Internet Connections
RTP	Real-time Transport Protocol
SBC	Single-board computers
SCTP	Stream Control Transmission Protocol
SDN	Software-defined networking
SDR	Software-defined radio
SIP	Session Initiation Protocol
TCP	Transmission Control Protocol
TNS	Time-Sensitive Networking
UAV	Unmanned aerial vehicle
UDP	User Datagram Protocol
W3C	World Wide Web Consortium
WebRTC	Web real-time communication
WLAN	Wireless local network
WMMS	WebRTC multimedia and monitoring station
WoT	Web of Things
XMPP	Extensible Messaging and Presence Protocol
**Indexes**	
*i*	*i*-th IoT datum
*j*	*j*-th IoT datum
ilc	input of logical channel
lc	logical channel level
olc	output of logical channel
*t*	transport level (output of transport protocol)
WRTC	WebRTC’s web logical channel (Data Channel)
WS	WebSocket web logical channel
**Symbols**	
*d*	end-to-end delay
$d^{lc}$	end-to-end delay measured at the logical channel level
$d^{t}$	end-to-end delay measured at the transport level
*D*	series of end-to-end delays
$D^{lc}$	series of end-to-end delays at the logical channel level
$D^{t}$	series of end-to-end delays at the transport level
DT	truncated series of end-to-end delays
$DT^{lc}$	truncated series of end-to-end delays at the logical channel level
$DT^{t}$	truncated series of end-to-end delays at the transport level
*t*	time
$t_{1}$	starting time
$t^{ilc}$	entry time into the logical channel
$t^{olc}$	reception time from the logical channel
$t^{t}$	reception time from the transport protocol
μ(D)	arithmetic mean of the time series D
μ(DT)	truncated arithmetic mean of the time series D
max(D)	maximum value in series D
max(DT)	truncated maximum value in series D
med(D)	median of the time series D
med(DT)	truncated median of the time series D
min(D)	minimum value in series D
min(DT)	truncated minimum value in series D
mod(D)	mode of the time series D
mod(DT)	truncated mode of the time series D
Q1(D)	lower quartile of the time series D
Q1(DT)	truncated lower quartile of the time series D
Q3(D)	upper quartile of the time series D
Q3(DT)	truncated upper quartile of the time series D
